# Privacy and Anonymity in the Information Society – Challenges for the European Union

**DOI:** 10.1100/tsw.2011.46

**Published:** 2011-03-01

**Authors:** Ioannis A. Tsoukalas, Panagiotis D. Siozos

**Affiliations:** European Parliament, Brussels, Belgium

**Keywords:** privacy, biometrics, information society, information and communication technologies, privacy by education, European Union, European Parliament

## Abstract

Electronic information is challenging traditional views on property and privacy. The explosion of digital data, driven by novel web applications, social networking, and mobile devices makes data security and the protection of privacy increasingly difficult. Furthermore, biometric data and radiofrequency identification applications enable correlations that are able to trace our cultural, behavioral, and emotional states. The concept of privacy in the digital realm is transformed and emerges as one of the biggest risks facing today's Information Society. In this context, the European Union (EU) policy-making procedures strive to adapt to the pace of technological advancement. The EU needs to improve the existing legal frameworks for privacy and data protection. It needs to work towards a “privacy by education” approach for the empowerment of “privacy-literate” European digital citizens.

## INTRODUCTION: THE EXPLOSION OF DIGITAL DATA

Electronic information constitutes a technological innovation by itself, one that is challenging people's understanding of the concepts of property and privacy[1]. These concepts, which appear to exist in every human society, are central to social and moral life, and provide a valuable prism for examining how cultures adapt to social and technological innovation. While in the physical world they are more or less well defined and stable over time, their evolution in the digital realm is much more volatile.

As more applications utilize the Internet and as, with the advent of the "Internet of Things" and nanotechnology, more of our everyday devices become interconnected, the size of digital information is exploding[2]. It is estimated that humanity, as a whole, created 150 billion gigabytes of data in the year 2005. Our current digital output stands at 800 billion gigabytes and is expected to pass 1.2 zettabytes at the end of this year[3] (i.e., $1.2 \times 10^{21}$ bytes or about *200 gigabytes per inhabitant of this planet*).

All these digital data have already exceeded our storage capability and are increasingly transforming the way we do policy, business, and science. As societies are becoming more dependent upon the widespread use of information and communication technologies (ICT), this immense flow – the phenomenon named by technologists as "big data – has the potential to spur innovation and to drive growth. However, it can also overflow our senses, clutter our societies, and dissolve our "private spheres".

As our use of the Internet and related technologies evolves, the protection of personal data is increasingly relevant[4]. Novel ways for widely distributing and sharing digital information appear every day, making data security and the protection of privacy even harder. ICT is becoming ubiquitous, businesses and citizens become more vulnerable, and privacy emerges as one of the biggest risks facing today's Information Society. Safeguarding all this information and giving consumers control and choice is a prerequisite for ensuring that any personal information that we share benefits citizens and consumers and drives innovation. Yet privacy is not only about risks, but also about opportunities.

The rapid growth of the "digital universe" has been caused mainly by the explosion of social networking, online video, digital photography, and mobile phones. Around 70% of the world's digital content is generated by individuals and most of these data are stored by large private companies on content-sharing websites such as YouTube. In fact, most of the innovative companies on the Internet are based on consumers willingly sharing their personal data. What we do online; what we shop for; what we like, dislike, or communicate; where we are and where we go; even our most intimate thoughts and feelings are accessible through the electronic trails we leave behind. All these massive personal data that the Internet companies collect allows them to provide valuable services and products to end users. Users do not seem to mind this exploitation of their private data. This is the price that they pay for all the amazing experiences that are offered to them.

## BIOMETRICS AS A CHALLENGE TO PRIVACY AND INDIVIDUALITY

However, all these vast digital data are nothing compared to the "biometric output" that is produced by our physical behavior as we work, travel, and socialize; as we live our everyday lives. Biometric data are not new; they have been used for more than 70 years (in the form of simple photos and description of physical traits) for securing travel documents. What is new is (1) the speed with which biometric data can be collected, stored, and exchanged by interoperable data systems and data miners; and (2) their correlation with information from which cultural, behavioral, and emotional data may be extracted[5]. Biometrics *per se* is not a problem. The way that the data are used, however, can be, giving base to citizens' concerns about the creation of "Big Brother" states. These conditions have delivered new challenges for intrusion on privacy and data protection. What is at stake is not only our *privacy*, but the notion of our *individuality*.

Governments trying to respond to terrorism and security threats consider biometrics as a very attractive tool[5]. Current applications of biometrics are multipurpose, ranging from regulation of asylum and migration to electronic patient records, access by personnel to sensitive areas, to flight security, and the fight against terrorism. However, some applications seem to be more far-fetched and employ the use of biometrics for disproportionate, imprecise, and invisible uses. Often, biometrics are used as an excuse for the creation of centralized databases, while many of the applications have been outsourced to private enterprises[6]. We have to bear in mind that today's technologies are perfectly able to support every possibility Orwell predicted this in his novel *1984*, giving us the capacity for real-time global surveillance.

The expanding use of biometrics gives rise to controversy. Until a few years ago, the European understanding of "biometrics" was based on a measurement of a given visible and unique physical feature of a person (such as a fingerprint, a hand print, an iris print). By now, this definition has been stretched to include invisible characteristics of a person, such as behavioral and emotional responses, as well as DNA samples and brain imaging[6]. The whole agenda of biometrics (from their definition to their application) seems to be driven by the U.S., while the EU, either as a whole or as individual member states, has made several concessions on matters of principal.

Biometric tools were originally intended to boost security and minimize risk for legitimate reasons[7], but the privacy trade-off for security reasons is being constantly challenged[8]. Biometrics, along with electronic IDs and RFID (radiofrequency identification) applications, are increasingly being used for trivial and controversial tasks, like recording the presence of children in school or certifying someone's age for buying alcohol[9]. Biometric applications can be developed by anyone and used for any purpose, raising important ethical questions and bringing novel threats to privacy protection.

In most EU member states, there is a trend for government agencies and corporate interests that use biometric documents to underestimate and downplay the technical disadvantages of biometrics and to advocate their widespread use, as a way of modernization[5]. However, this indiscriminate deployment of biometrics aggravates anxiety as to their disproportionate use, "mission creep", and potential infringements of citizens' privacy[6].

## THE NOTION OF PRIVACY IN THE DIGITAL REALM

*So, "is privacy dead", as some industry experts have declared? Have we lost the right to control how our personal information is used? Are digital data and biometric devices the building blocks of Panopticon societies?*

The answer seems to be a delicate and ever-changing balance between privacy and security. One that is constantly challenged by technological innovation; yet technological innovation seems currently to be the only thing that can set this balance straight again. The protection of privacy is a constant arms race, the "cold war" of the 21st century.

Undeniably, the digital world evidently offers great potential for innovation, but it is critical that we get the privacy issue right. Governments, companies, and citizens have to be able to make the right choices. After all, centralized control, protectionism, and the control of information flows are very attractive to every power system, either public or private.

At a basic level, privacy in the online and the offline world are similar: citizens or consumers have a right to privacy and the proper use of their personal data. They expect that public authorities and companies will collect, analyze, share, and safeguard their data properly. In this respect, digital communities do not differ much from physical communities: they face similar risks, must take similar precautions, and must conform to the same principles.

Nevertheless, the online world introduces additional complexity. Digital data are collected in manners that we often fail to understand. The characteristics of the digital realm tend to reinforce the disconnectedness between *cause* and *effect*: the actions of a digital citizen are immaterial, undetectable, and nonidentifiable[10]. This potential for *impersonal crime* (with no visible or known victim), as well as the playful nature of the digital world, is challenging the traditional ethical theories of our offline societies[11]. After all, how responsible do our children feel when killing numerous digital "enemies" in a computer game?

## EUROPEAN UNION AND PRIVACY-LITERATE SOCIETIES

In this context, our policy-making procedures seem unable to handle the transition to the digital era. Our political, social, and educational institutions do not always possess the necessary flexibility to adapt to the rapid rhythms of digital cosmogony. The pace of technological advances outrivals the ability of legislators to address the issues that are raised and to introduce measures to safeguard citizens. After all, in most cases, technologies were designed as tools for solving problems of quantity ("faster, cheaper, more") and not of quality. And more often than not, technological innovations take policy makers as well as business strategists by surprise.

The EU has accepted that privacy is a precondition for many other fundamental rights and, therefore, the right to privacy has been enshrined in Article 8 of the European Convention on Human Rights (ECHR). The EU, however, needs to improve the existing legal frameworks for privacy and data protection. It is in need of a concrete framework for the production of legislation, recommendations, and best practices for biometric and personal data in regards to innovation and privacy. And this has to be adopted and enforced with uniformity, within the EU, in line with European policy goals and values.

The European Commission, the member states, the private sector, and consumer groups have to work together to clarify the relationship between users and their online personal data profiles (e.g., [6,7,12]). These recommendations can ensure that consumers have control over their personal data, helping to increase innovation and promote a robust and healthy digital ecosystem. In this context, the EU is supporting valuable research projects, such as RISE (http://www.riseproject.eu) and HIDE (http://www.hideproject.org/), which seek to raise awareness and to promote global dialogue and practical recommendations regarding the use of biometric and private data.

The European Parliament should be adequately informed and able to evaluate legislative options for addressing and safeguarding the EU citizens' liberties. It should use the power given to it by the Lisbon Treaty and proceed in the direction of negotiating, on behalf of the European citizens, a framework on data protection (including biometric data) that will reflect the European values and will be able to protect all EU citizens, either on EU territory or not. The capacity of the EU to balance between multiple and possibly contradictory expectations, such as to deliver security, ensure democracy and welfare, and protect citizens' fundamental rights, is being put to the test[12].

The EU and various private and social stakeholders promote the use of the "privacy by design" concept, a methodology that provides for privacy-related issues all along the life cycle of a product or service, from its inception to its decommissioning[13,14,15]. While this concept is highly useful from an engineering point of view, EU legislators should approach it from a social engineering point of view, and extend it to include "*privacy by education*". The only viable defense against privacy risks is the empowerment of digital citizens, which is achieved through information and education regarding digital risks, rights, and obligations. Citizens, students, teachers, future engineers, and programmers, as well as policy makers and legislators, should all be educated regarding privacy-related issues and the implications of their online and offline behavior.

The European response to privacy issues should be the establishment of open, participatory, and "privacy-literate" societies, which are aware of the risks and are empowered, both technologically and through self-conscious behaviors. We have to bear in mind that the EU is no more just an economic union based on free market, but a union of thriving democracies with social and human rights at its core. Europe should once again be established as the global leader, not only in technological issues, but also in ethical standards.
